# Detection of *Acanthamoeba* spp. using carboxylesterase antibody and its usage for diagnosing *Acanthamoeba*-keratitis

**DOI:** 10.1371/journal.pone.0262223

**Published:** 2022-01-05

**Authors:** Min-Jeong Kim, Ki-Back Chu, Hae-Ahm Lee, Fu-Shi Quan, Hyun-Hee Kong, Eun-Kyung Moon

**Affiliations:** 1 Department of Biomedical Science, Graduate School, Kyung Hee University, Seoul, Republic of Korea; 2 Medical Research Center for Bioreaction to Reactive Oxygen Species and Biomedical Science Institute, School of Medicine, Graduate school, Kyung Hee University, Seoul, Republic of Korea; 3 Department of Medical Zoology, Kyung Hee University School of Medicine, Seoul, Republic of Korea; 4 Department of Parasitology, Dong-A University College of Medicine, Busan, Republic of Korea; Wayne State University School of Medicine, UNITED STATES

## Abstract

Contact lens usage has contributed to increased incidence rates of *Acanthamoeba* keratitis (AK), a serious corneal infection that can lead to blindness. Since symptoms associated with AK closely resemble those incurred by bacterial or fungal keratitis, developing a diagnostic method enabling rapid detection with a high degree of *Acanthamoeba*-specificity would be beneficial. Here, we produced a polyclonal antibody targeting the carboxylesterase (CE) superfamily protein secreted by the pathogenic *Acanthamoeba* and evaluated its diagnostic potential. Western blot analysis revealed that the CE antibody specifically interacts with the cell lysates and conditioned media of pathogenic *Acanthamoeba*, which were not observed from the cell lysates and conditioned media of human corneal epithelial (HCE) cells, *Fusarium solani*, *Staphylococcus aureus*, and *Pseudomonas aeruginosa*. High titers of *A*. *castellanii*-specific antibody production were confirmed sera of immunized mice via ELISA, and these antibodies were capable of detecting *A*. *castellanii* from the cell lysates and their conditioned media. The specificity of the CE antibody was further confirmed on *A*. *castellanii* trophozoites and cysts co-cultured with HCE cells, *F*. *solani*, *S*. *aureus*, *and P*. *aeruginosa* using immunocytochemistry. Additionally, the CE antibody produced in this study successfully interacted with 7 different *Acanthamoeba* species. Our findings demonstrate that the polyclonal CE antibody specifically detects multiple species belong to the genus *Acanthamoeba*, thus highlighting its potential as AK diagnostic tool.

## Introduction

*Acanthamoeba* spp. are free-living amoeba widely distributed in nature and are the causative agents of *Acanthamoeba* keratitis (AK) and granulomatous amebic encephalitis (GAE) in humans [1–3]. Currently, *Acanthamoeba* is classified into 20 genotypes (T1-T20) based on their 18S ribosomal DNA gene sequence, with T4 being the genotype most frequently associated with AK and GAE [4–6]. In addition, they can also be categorized into groups I through III based on their endocyst and ectocyst morphological features described by Pussard and Pons, with a majority of the strains responsible for AK belonging to group II [7–9]. Global incidence rate of AK continues to rise and the major risk factor associated with it has been identified to be contact lens usage [10–12]. However, non-contact lens wearers can also be susceptible to acquiring AK upon corneal damage or exposure to *Acanthamoeba*-contaminated water [3,10,13]. Initial signs of AK were reported to be generally unilateral, which begins at the epithelial layer and gradually progressing towards the corneal stroma [13,14]. Unfortunately, clinical manifestations of AK are strikingly similar to corneal infections incurred by other pathogens, and differentiating bacterial or fungal keratitis from that of amoebic origin is difficult [2,14–16]. Since delayed or misdiagnosis of AK can lead to serious eye damage that can result in permanent vision loss a diagnostic test enabling rapid and accurate detection of AK is urgently needed [2,3,16].

Currently available techniques for diagnosing AK are confocal microscopy, polymerase chain reaction (PCR), histopathological examination, and microbiological culture [2,14,16–18]. While these have been useful, there are several limitations associated with each of the aforementioned diagnostic methods. Confocal microscopy can identify the distinct double-wall of *Acanthamoeba* cysts but fails to accurately distinguish leukocytes from *Acanthamoeba* trophozoites [17–19]. PCR-based assays are highly sensitive and allow rapid diagnosis, but they cannot differentiate between live *Acanthamoeba* and dead *Acanthamoeba* in clinical samples [17,18,20,21]. Microbiological culture is another highly sensitive diagnostic method, but lengthy result acquisition time limits their usage [2,17,18]. Histopathological examinations or multiple staining procedures are the most reliable methods for AK diagnosis at present, but they require corneal scrapings from patients which inflict a tremendous amount of pain [13,16,17]. These culture-based methods, though highly accurate, are time-consuming and test results cannot be provided immediately. Given these circumstances, developing a non-invasive AK diagnostic method that accurately and rapidly identifies *Acanthamoeba* spp. without the need for specialized equipment would be promising.

Recently, several studies have reported antibody-based diagnostic methods for AK. A polyclonal inosine-uridine preferring nucleoside hydrolase (IPNH) antibody specifically detected *A*. *castellanii* trophozoite, while peptide antibody targeting the chorismate mutase of *Acanthamoeba* spp. successfully detected both trophozoites and cysts of *A*. *castellanii* [22,23]. Five monoclonal antibodies (AMEC1, AMEC2, AMEC3, MTAC1, and MTAC3) were generated from hybridoma cell lines of mice immunized with live mixtures of *A*. *castellanii* trophozoites and cysts, which successfully interacted with cysts or trophozoites of *A*. *castellanii*, *A*. *polyphaga*, *A*. *lenticulata*, and *A*. *culbertsoni* [24]. Due to the ubiquitous nature of *Acanthamoeba* spp., *Acanthamoeba*-specific IgG and IgA antibodies were reported to be present in sera and tears of healthy individuals [25]. Serum samples from patients have previously been used to detect *Balamuthia mandrillaris* and *Acanthamoeba* spp. by indirect immunofluorescence antibody (IFA) staining [26]. As the diagnostic method based on antibodies has been developed, the importance of antibodies for diagnosing AK is being emphasized. Yet, commercialized antibody-based AK diagnosis remains unavailable and additional research needs to be conducted.

Carboxylesterase (CE) is an enzyme that hydrolyzes esters, thioesters, and amine functional groups on a compound [27–29]. CE is widespread and has extensive substrate specificity in various mammalian species [27,30,31]. Molecular hydrolysis exerted by CE is known to be involved in drug and detoxification mechanisms [27,31]. Most CEs are intracellular proteins, but some CEs are secreted by cells [31]. In our previous study, we identified 34 increased proteins and 7 qualitatively increased protein expressions in the pathogenic strain of *Acanthamoeba* compared to the non-pathogenic *Acanthamoeba*, with CE superfamily protein being one of them [32]. Here, we produced a full-length polyclonal antibody against the CE secreted from pathogenic *Acanthamoeba* and evaluated its diagnostic potential. The specificity of the CE antibody was investigated by western blot, immunocytochemistry, and ELISA analysis. Our findings demonstrate that the highly specific nature of *Acanthamoeba* CE antibody can be used for AK diagnosis.

## Materials and methods

### Cell culture

*Acanthamoeba castellanii* Castellani was obtained from the American Type Culture Collection (ATCC 30868), and six different *Acanthamoeba* species (*A*. *castellanii* Neff, *A*. *hatchetii*, *A*. *polyphaga*, *A*. *culbertsoni*, *A*. *royreba*, and *A*. *healyi*) were kindly provided by Prof. Ho-Joon Shin at Ajou University (Suwon, Republic of Korea). *Acanthamoeba* trophozoites were cultured in Peptone-Yeast- Glucose (PYG) media at 25°C for 5 days, and the cells and culture media were harvested. *Acanthamoeba* cysts were induced in encystment medium (95 mM NaCl, 5 mM KCl, 8 mM MgSO_4_, 0.4 mM CaCl_2_, 1 mM NaHCO_3_ and 20 mM Tris-HCl, pH 9.0) at 25°C. The morphological transformation of the cells into mature cysts was confirmed using light microscopy. *Fusarium solani*, *Pseudomonas aeruginosa*, and *Staphylococcus aureus* were obtained from the Korea Centers for Disease Control & Prevention (NCCP 32678, NCCP 16091, and NCCP 15920). *F*. *solani* was incubated on Sabouraud Dextrose (SD) media at 25°C for 3 days, and *P*. *aeruginosa* and *S*. *aureus* were cultured on Brain Heart Infusion (BHI) media at 37°C for 1 day. Cells and culture media were collected after incubation. Human corneal epithelial (HCE) cells were obtained from the American Type Culture Collection (PCS-700–010). HCE cells were cultured at 37°C with 5% CO_2_ in endothelial cell growth medium kits (KGM BulletKit) (Lonza, Portsmouth, NH), and the cells and culture media were collected.

### Cloning, expression, and antibody generation

The open reading frame of carboxylesterase (CE) superfamily protein of *A*. *castellanii* (GenBank MW683236) was amplified (forward primer: 5’-GAAT GGATCCATGCATCGCTCTACCCT-3’, reverse primer: 5’-ACTAGAATTCCTAGACAGAGATGGAGTTCCAC-3’) and cloned into a pGEX 4T-3 GST-tagged expression vector (GE Healthcare, Buckinghamshire, UK). GST-fused CE recombinant protein was induced in *Escherichia coli* BL21 (DE3) with 1 mM isopropyl-β-D-thiogalactopyranoside (IPTG) at 37°C for 4 h. The expressed protein was resolved on a 10% sodium dodecyl sulfate-polyacrylamide gel electrophoresis (SDS-PAGE) gel and visualized with Coomassie brilliant blue staining. The protein was purified using the EzWay™ PAG Protein Elution Kit V2 (Komabiotech, Seoul, Korea). Purified proteins were concentrated with an Amicon Ultra-4 centrifugal filter device (Merck Millipore, Burlington, MA, USA). Six-week-old male BALB/c mice (n = 2 per group) purchased from KOATech (Pyeongrak, Korea) were used for antibody generation. Purified CE protein (50 μg) was mixed with an equal volume of Freund’s complete/incomplete adjuvant (Sigma-Aldrich, St. Louis, MO, USA) and subcutaneously inoculated into mice. Mice were immunized a total of 3 times at 3 week intervals as described previously [22]. Sera were collected 1 week after the final immunization. Experimental protocols have been approved and all experiments were performed following the Kyung Hee University’s Institutional Animal Care and Use Committee (IACUC) guidelines (permit No. KHSASP-20-326).

### Western blot assay

Antibody verification was performed using western blot. Whole cell lysates and conditioned media of HCE cells, *A*. *castellanii*, *F*. *solani*, *S*. aureus, and *P*. *aeruginosa* were prepared. Protein samples were confirmed on 10% SDS-PAGE, and transferred to a nitrocellulose membrane. Membranes were blocked with 5% skim milk in TBST (25 mmol/L Tris base, 150 mmol/L NaCl, and 0.1% Tween 20) for 2 h and incubated overnight at 4°C with the CE antibody (1:1,000). Membranes were washed with TBST and probed with horseradish peroxidase (HRP)-conjugated anti-mouse IgG (Sigma-Aldrich, St. Louis, MO, USA) (1:5,000) for 1 h at RT. Bands were developed using Clarity Enhanced Chemiluminescence (ECL) reagent (Thermo Fisher, Waltham, MA, USA) on x-ray films in the darkroom.

### Enzyme-linked immunosorbent assay (ELISA)

*Acanthamoeba*-specific antibody responses were determined by ELISA. Antigens and sera were serially diluted (10^2^ to 10^−10^ μg/μl and 1:50 to 1:50,000, respectively) to assess optimal detection concentrations. Briefly, 96-well plates were coated with antigens in carbonate coating buffer (0.1 M Sodium Carbonate, pH 9.5) overnight at 4°C. Plates were washed 3 times with PBST and blocked with 0.2% gelatin for 2 h at 37°C. CE antibodies diluted in PBS were inoculated into wells and incubated at 37°C for 1 h. HRP-conjugated anti-mouse IgG (Sigma-Aldrich, St. Louis, MO, USA) was added at 1:1,000 dilution in PBS and incubated at 37°C for 1 h. O-phenylenediamine (OPD) substrate was purchased from Zymed (San Francisco, CA, USA) and dissolved in citrate-phosphate buffer (pH 5.0) with 0.03% H_2_O_2_. The optical density values at 450 nm were read using EZ Read 400 microplate reader (Biochrom Ltd., Cambridge, UK). The sera of the unimmunized mice were used as a negative control.

### Immunocytochemistry (ICC)

HCE cells (3×10^5^ cells) were cultured on sterile cover glass in a 6-well plate. The following day, they were co-cultured with *A*. *castellanii* trophozoites (5×10^5^ cells), and cysts (5×10^5^ cells) for 5 h at 37°C with 5% CO_2_. *F*. *solani*, *P*. *aeruginosa*, and *S*. *aureus* were cultured in liquid broths until early exponential phase (OD_600nm_ = 0.8), which were subsequently co-cultured with HCE cells and *A*. *castellanii* for 1 h. After washing with PBS, the cells were fixed with ice-cold 100% methanol for 5 min at RT. Cells were repeatedly washed and permeabilized with PBST (1 X PBS containing either 0.1% Tween 20) for 10 min at RT. Afterward, cells were washed and subsequently blocked using blocking buffer (1% bovine serum albumin and 22.52 mg/ml glycine in PBST) for 30 min at RT. Cells were incubated overnight at 4°C with CE antibody (1:200) in blocking buffer and probed with fluorescein isothiocyanate (FITC)-conjugated anti-mouse IgG (1:400) for 1 h at RT. After repeated washing, cells were stained with VECTASHIELD mounting medium (Abcam, Burlingame, CA, USA) and observed under a fluorescent microscope (Leica DMi8, Wetzlar, Germany).

### Statistical analysis

Student’s *t*-test were performed using GraphPad Prism version 5 (San Diego, CA, USA). Data are expressed as mean±SD. Statistical significance between the means of groups were denoted using an asterisk. *P* values less than 0.05 was considered statistically significant (*P < 0.05, ** *P* < 0.01, and *** *P* < 0.001).

## Results

### Generation of CE polyclonal antibody

**Fig 1:**
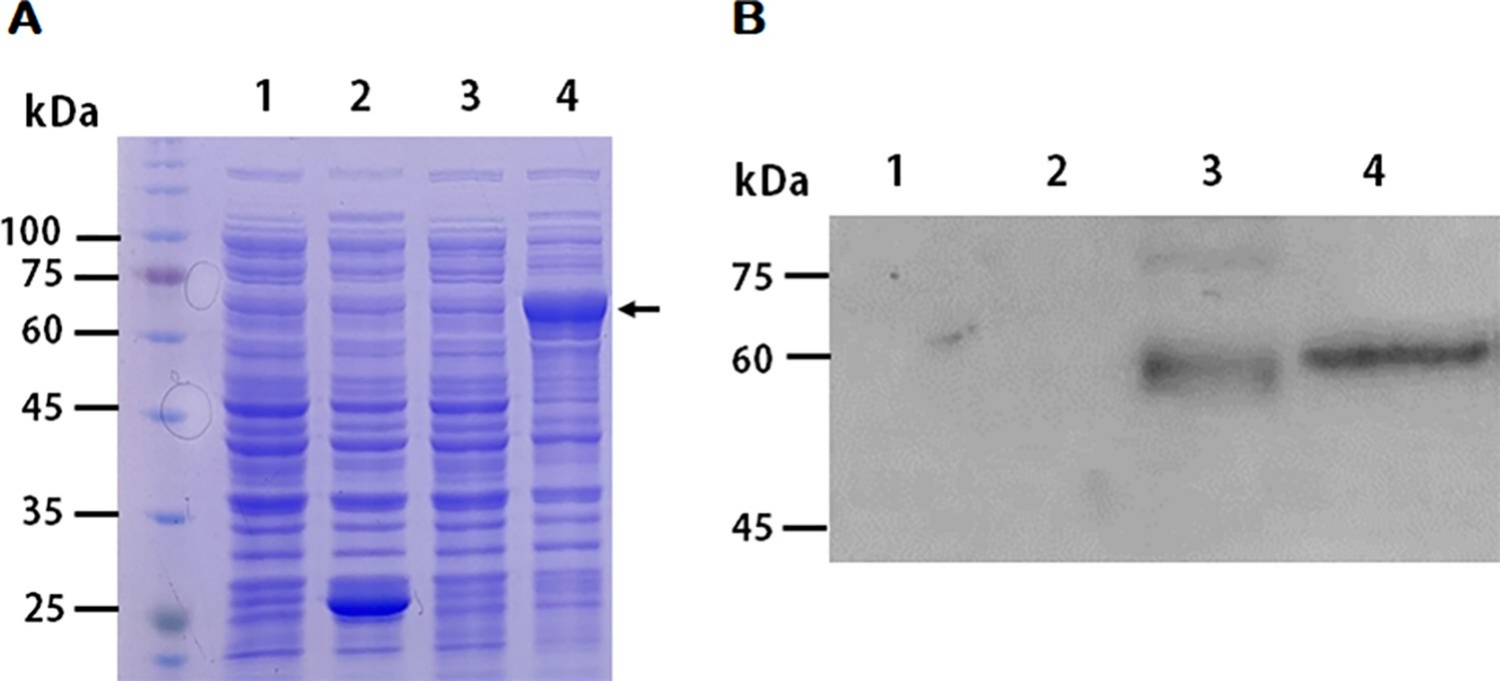
Generation of full-length polyclonal antibody against CE. (A) Expression of the GST-CE fusion protein in *E*. *coli* BL21(DE3) was confirmed by SDS-PAGE. M: Protein size marker, Lane 1: pGEX-4T-3 before induction, Lane 2: pGEX-4T-3 after induction, Lane 3: pGEX-4T-3-CE before induction, Lane 4: pGEX-4T-3-CE after induction (arrow). (B) Western blot analysis was performed to examine the generation of CE antibodies. Generated anti-CE antibody was used to identify CE proteins in cell lysate (50 μg) and conditioned media (15 μg) of HCE cells and *A*. *castellanii*. Lane 1: Cell lysate of HCE cells, Lane 2: Conditioned media of HCE cells, Lane 3: Cell lysate of *A*. *castellanii*, Lane 4: Conditioned media of *A*. *catellanii*.

The full-length open reading frame of carboxylesterase (CE) obtained from pathogenic *A*. *castellanii* consists of 1,602 bp and encodes 533 amino acids with a calculated mass of 58.63 kDa. Peptide analysis using SignalP 4.1 predicted the signal peptide and the transmembrane domain to be located at the amino acid positions 1 to 22 and 7 to 29, respectively. The CE superfamily protein secreted by the pathogenic *A*. *castellanii* was cloned into a pGEX-4T-3 vector, and GST-CE fusion protein was expressed with 1 mM IPTG. As illustrated below (Fig 1A), protein expression of 26 kDa GST protein (lane 2) and 85 kDa GST-CE fusion protein (lane 4, arrow) was well-established in *E*. *coli*. The GST-CE fusion protein was purified and used to immunize mice for polyclonal antibody generation. Successful generation of CE antibody was confirmed by western blot analysis using cell lysates and conditioned media of human corneal epithelial (HCE) cells and *A*. *castellanii* (Fig 1B). The CE antibody did not react to cell lysates and conditioned media of HCE cells (lanes 1 and 2), while specifically reacted to those of *A*. *castellanii* (lanes 3 and 4).

### IgG antibody responses to *Acanthamoeba* antigen

**Fig 2:**
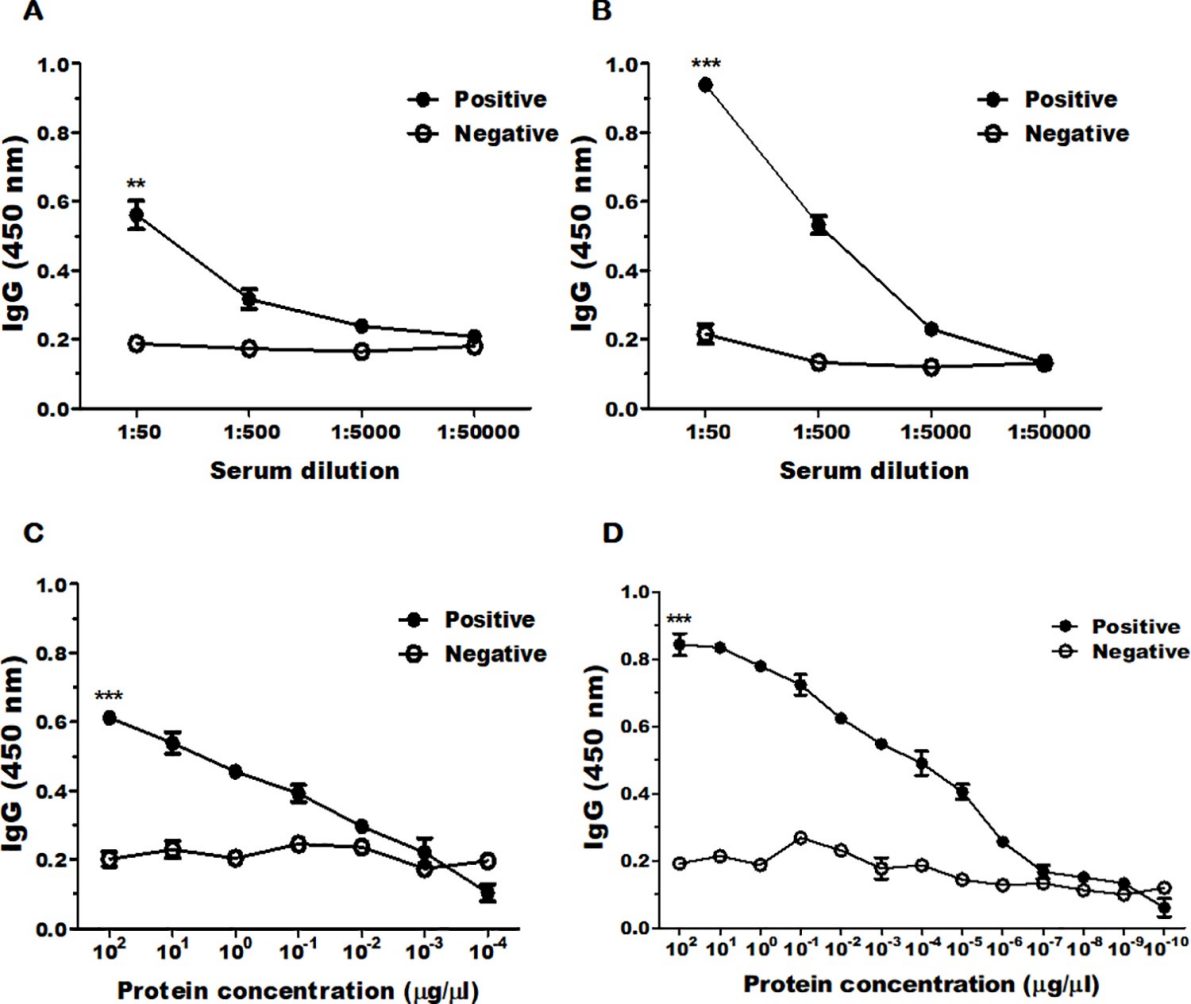
CE of *A*. *castellanii*-specific IgG response. Serially diluted CE antibodies were titrated using cell lysates (A) and conditioned media (B) of *A*. *castellanii* by ELISA. The sensitivity of CE antibodies was also determined using serially diluted cell lysates (C) and conditioned media (D) of *A*. *castellanii*. Positive; immune sera, Negative; naïve (unimmunized) sera. Asterisks denote statistically significant differences (** *P* < 0.01 and *** *P* < 0.001) between positive and negative serum. Data are expressed as mean±SD.

ELISA was performed to confirm *Acanthamoeba*-specific antibody response. Mice immunized with the CE of *A*. *castellanii* produced high titer of antibodies, which were serially diluted to assess optimal detection concentration against *Acanthamoeba* cell lysates (Fig 2A) and conditioned media (Fig 2B). When compared to the negative control (unimmunized sera), differences in optical density values were negligible to marginal at 1:50,000 and 1:5,000 dilutions, respectively. However, noticeable differences were observed at dilutions of 1:50 and 1:500. To assess the sensitivity of the CE antibody, antigens acquired from cell lysate (Fig 2C) and the conditioned media (Fig 2D) were serially diluted from 10^2^ μg/μl to approximately 10^−10^ μg/μl. The detection limit of CE-specific antibody against cell lysate and conditioned media antigens were 10^−1^ μg/μl and 10^−5^ μg/μl, respectively, thus indicating the highly sensitive nature of the CE antibody of *A*. *castellanii*.

### Specificity of full-length polyclonal CE antibody

**Fig 3:**
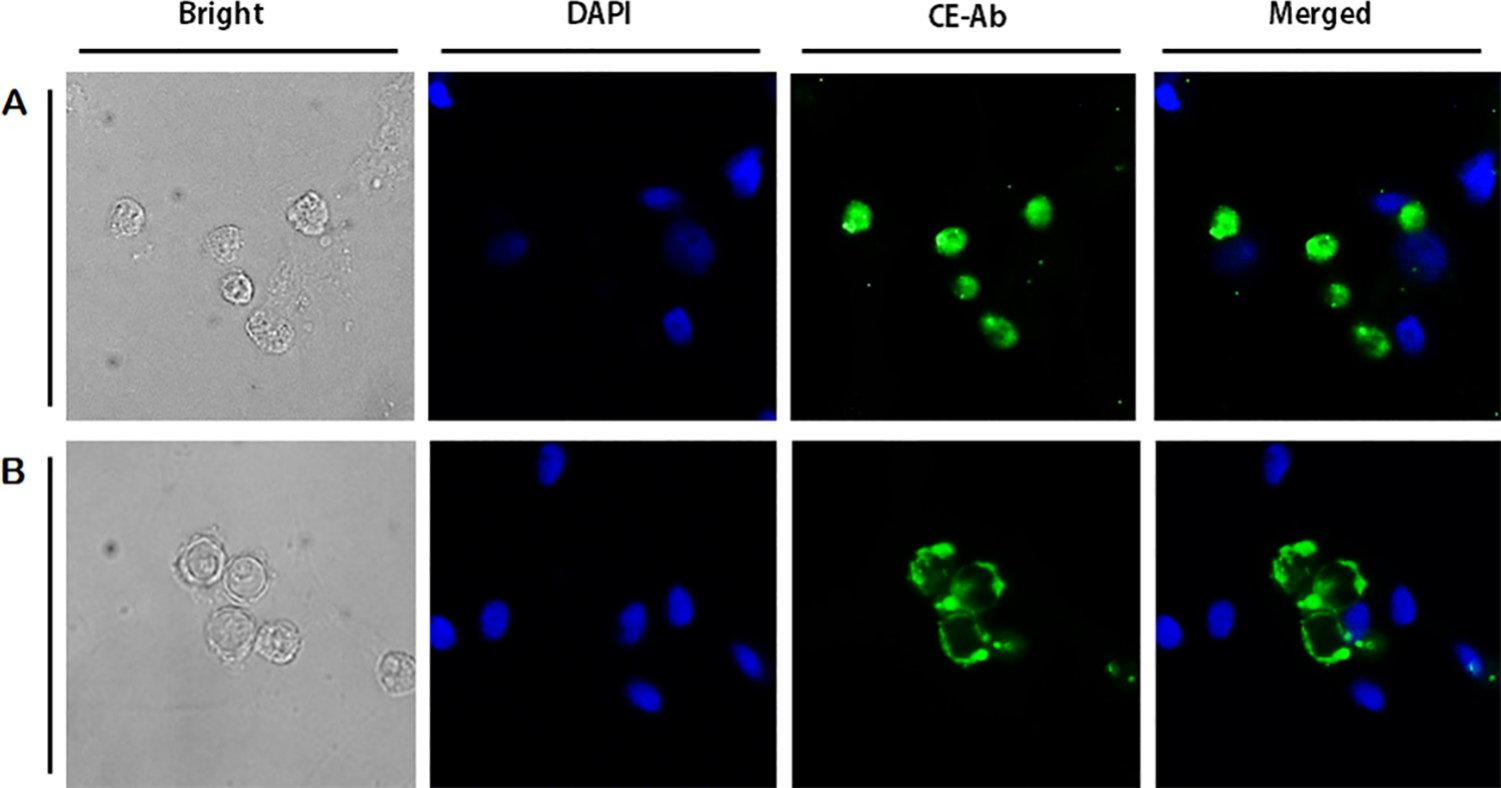
Immunocytochemistry using anti-CE antibodies. HCE cells and *A*. *castellanii* trophozoites (A) and cysts (B) were co-cultured for 5 h and observed under a fluorescent microscope. Bright-field, DAPI staining (blue), CE antibody combined with FITC-conjugated secondary antibody (green), and merged images were acquired at 400x magnification.

To confirm the CE antibody specificity, immunocytochemistry was performed using HCE cells co-cultured with *A*. *castellanii* trophozoites and cysts (Fig 3). The nuclei of HCE cells were stained with DAPI (blue), but CE labeled with FITC fluorophore (green) could not be detected on the cell surface. On the contrary, strong interaction between trophozoites (Fig 3A) and cysts (Fig 3B) of *A*. *castellanii* with the CE antibody (green) was observed. Since the size of *Acanthamoeba* nuclei are much smaller than those of HCE cells, DAPI-stained nuclei of *Acanthamoeba* were faintly visible.

### Sequence homology analysis and confirming CE-specificity of the antibody

**Fig 4:**
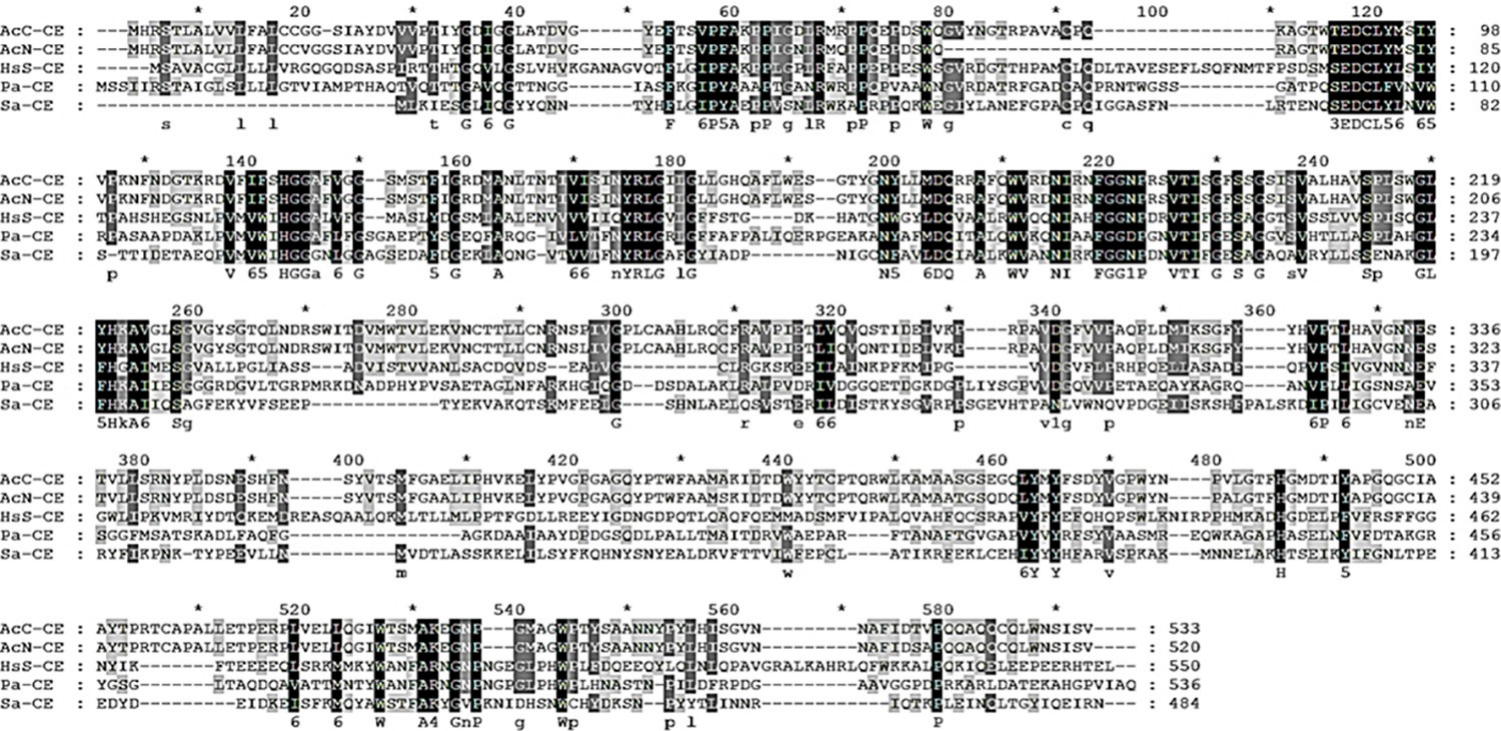
Multiple alignments of the CE amino acid sequences. Alignments of CE amino acid sequences of *A*. *castellanii* Castellani (AcC-CE, MW683236), *A*. *castellanii* Neff (AcN-CE, XP_004337797.1), *Homo sapiens* Sapiens (HsS-CE, AAB03611.1), *P*. *aeruginosa* (Pa-CE, WP_079867724.1), and *S*. *aureus* (Sa-CE, NGC32462.1) were compared. Multiple amino acid sequence alignment was produced using the CLUSTAL_X version 2.1. Conserved regions were denoted with black shading.

To further confirm that the CE of *Acanthamoeba* was highly specific and useful for AK diagnosis, amino acid sequences of CE of *A*. *castellanii* Castellani were compared with CE protein of *A*. *castellanii* Neff, *Homo sapiens* Sapiens, *P*. *aeruginosa*, and *S*. *aureus* (Fig 4). Amino acid sequence homology results revealed that CE of *A*. *castellanii* Castellani showed 94.19%, 27.92%, 29.11%, and 34.31% similarity with that of *A*. *castellanii* Neff, *H*. *sapiens* Sapiens, *P*. *aeruginosa*, and *S*. *aureus*, each respectively (Table 1). Because the CE gene sequence of *F*. *solani* was not reported in the GenBank database, sequence homology comparison with this group could not be performed.

**Table 1:** Identities of CE amino acid sequences among different organisms.

Name	Accession No.	Length (aa)	Identities (%)
AcC-CE	MW683236	533	100
AcN-CE	XP_004337797.1	520	94.19
HsS-CE	AAB03611.1	550	27.92
Pa-CE	WP_079867724.1	536	29.11
Sa-CE	NGC32462.1	484	34.31

**Fig 5:**
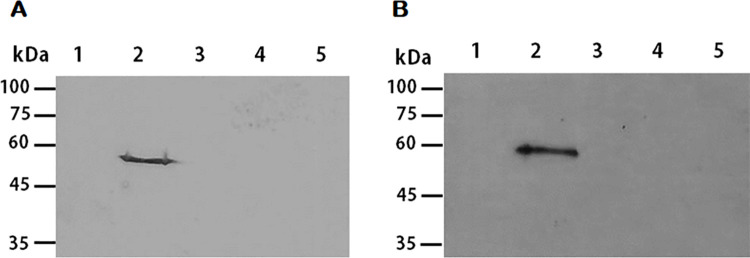
Western blot analysis of specific antibody response to CE protein. Western blot analysis was performed to specifically detect CE proteins of *A*. *castellanii* using anti-CE antibodies. Cell lysates (A) and conditioned media (B) from different organisms were used for western blot analysis. Lane 1: HCE cells, Lane 2: *A*. *castellanii*, Lane 3: *F*. *solani*, Lane 4: *S*. *aureus*, Lane 5: *P*. *aeruginosa*.

**Fig 6:**
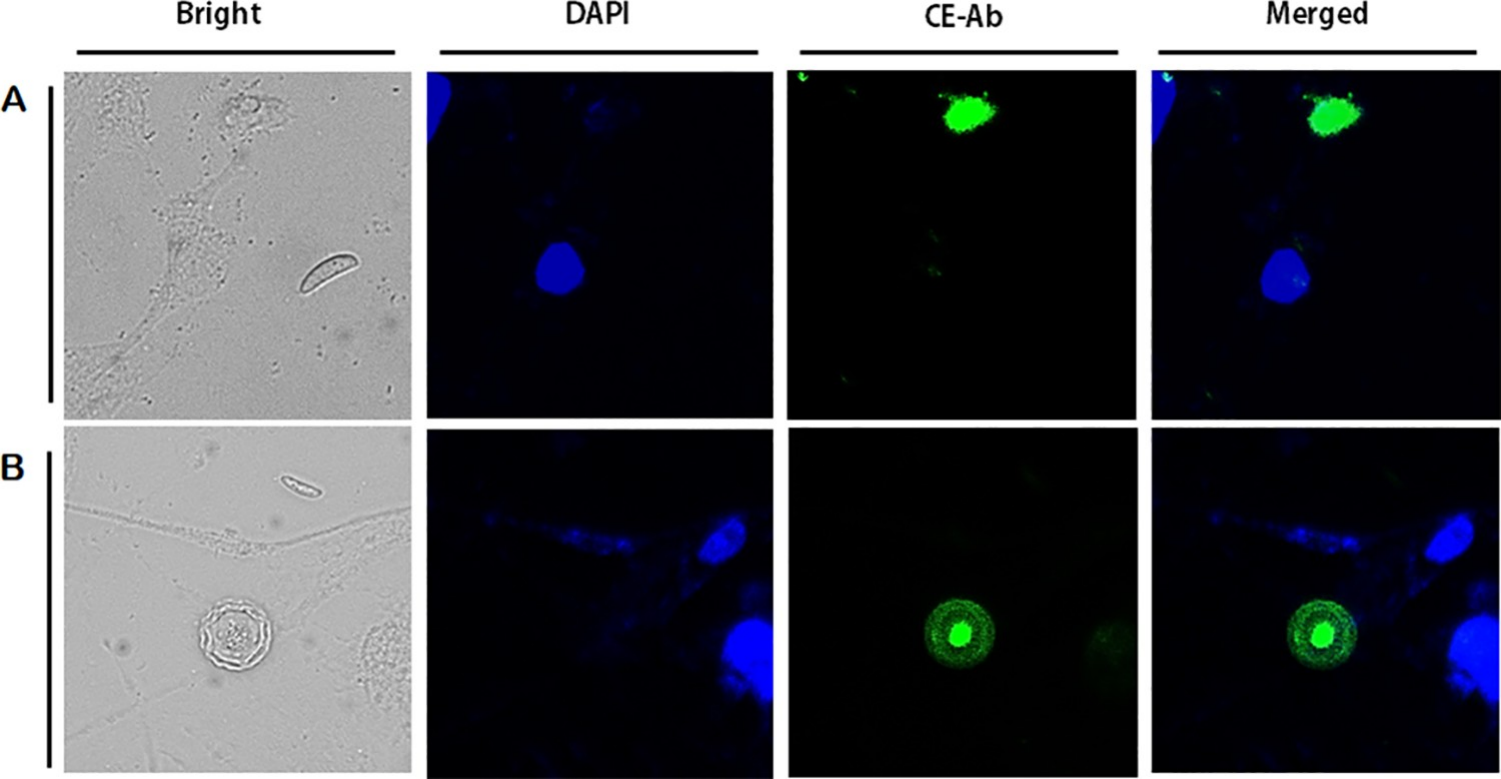
Immunocytochemistry using anti-CE antibodies. HCE cells and *A*. *castellanii* trophozoites (A) and cysts (B) were co-cultured for 4 h. *F*. *solani*, *S*. *aureus*, and *P*. *aeruginosa* were inoculated into the cultures and incubated for 1h. The co-cultured cells were observed under a fluorescent microscope. Bright-field, DAPI staining (blue), CE antibody combined with FITC-conjugated secondary antibody (green), and merged images were acquired at 400x magnification.

CE antibody specificity was further evaluated by confirming its reactivity with multiple causative agents of keratitis. Western blot analysis was performed using cell lysates and conditioned media of HCE cells, *A*. *castellanii*, *F*. *solani*, *S*. *aureus*, and *P*. *aeruginosa* (Fig 5). The CE antibody only reacted with cell lysate (Fig 5A) and conditioned media (Fig 5B) of *Acanthamoeba*, while interactions with the cell lysates and conditioned media of HCE cells, *F*. *solani*, *S*. *aureus*, *P*. *aeruginosa* were not observed. This result showed that the CE antibody of *A*. *castellanii* was able to specifically detect trophozoites and cysts of pathogenic *A*. *castellanii*. For further verification of CE antibody specificity, immunocytochemistry was performed using HCE cells co-cultured with *F*. *solani*, *P*. *aeruginosa*, *S*. *aureus*, and *A*. *castellanii* trophozoites and cysts (Fig 6). As expected, strong CE antibody interactions were only observed from *A*. *castellanii* trophozoites (Fig 6A) and cysts (Fig 6B).

### The response of CE antibody to different strains of *Acanthamoeba* spp.

**Fig 7:**
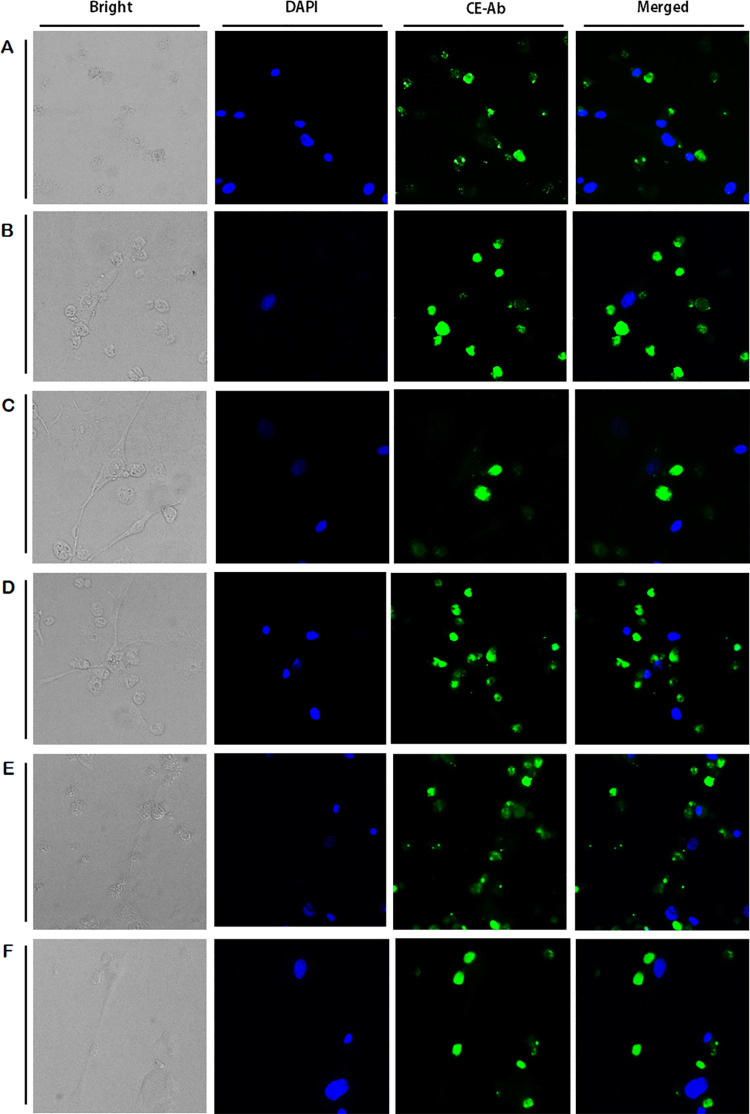
Immunocytochemistry of six reference *Acanthamoeba* spp. using CE antibodies. HCE cells and other six reference *Acanthamoeba* spp. trophozoites were co-cultured for 5 h and observed under a fluorescent microscope. Bright-field, DAPI staining (blue), CE antibody combined with FITC-conjugated secondary antibody (green), and the merged image were acquired at 400x magnification. A: *A*. *castellanii* Neff, B: *A*. *hatchetti*, C: *A*. *polyphaga*, D: *A*. *culbertsoni*, E: *A*. *royreba*, F: *A*. *healyi*.

Because AK can be caused by several members of the genus *Acanthamoeba*, immunocytochemistry was performed using HCE cells with 6 additional *Acanthamoeba* species belonging to the morphological group II (*A*. *castellanii* Neff, *A*. *hatchetti*, and *A*. *polyphaga*) and III (*A*. *culbertsoni*, *A*. *royreba*, and *A*. *healyi*). Successful binding of the CE antibody to trophozoites of all 6 species was observed (Fig 7A–7F). These results showed that the CE antibody generated in this study is capable of detecting various species of *Acanthamoeba* and immunocytochemistry as being a more sensitive method than western blotting for *Acanthamoeba* detection.

## Discussion

In this study, we evaluated the possibility of quick and differential diagnosis of AK using an antibody-based technique that can specifically identify multiples species belonging to the genus *Acanthamoeba*. We produced a full-length polyclonal antibody against the secretory CE protein of *A*. *castellanii*. The antibody generated in this study failed to interact with HCE cells, *F*. *solani*, *S*. *aureus*, and *P*. *aeruginosa* but successfully detected multiple members of the *Acanthamoeba*, thus highlighting its species specificity. It is important to note that the CE antibody demonstrated in our study specifically detects only *Acanthamoeba* spp. even when other keratitis-causing microorganisms were present, as illustrated in our immunocytochemistry results (Fig 6).

We have previously reported the diagnostic potential of *Acanthamoeba*-specific antibodies targeting the inosine-uridine preferring nucleoside hydrolase and chorismate mutase [22,23]. While these studies revealed that antibody-based detection method can specifically identify *A*. *castellanii* and differentiate it from other microbial causes of keratitis, our previous works did not investigate their diagnostic potential against other *Acanthamoeba* species. To date, 8 *Acanthamoeba* species were identified to be capable of causing keratitis and these are as follows: *A*. *castellanii*, *A*. *polyphaga*, *A*. *royreba*, *A*. *culbertsoni*, *A*. *hachetti*, *A*. *griffin*, *A*. *quina*, and *A*. *lugdunensis* [2,33–35]. As such, we investigated the interaction of the CE antibody with *A*. *castellanii* Castellani (group II), *A*. *castellanii* Neff (group II), *A*. *hatchetti* (group II), *A*. *polyphaga* (group II), *A*. *culbertsoni* (group III), *A*. *royreba* (group III), and *A*. *healyi* (group III) which were not addressed in our earlier works. Immunocytochemistry results revealed that the CE antibody reacted with multiple *Acanthamoeba* spp. (Fig 7), thereby confirming that the antibody used in the current study was highly specific for *Acanthamoeba*. Notably, the CE antibody also reacted with the GAE-inducing *A*. *healyi*. Current GAE diagnosis requires histological examinations using tissues and cerebrospinal fluids [36]. To this extent, the CE antibody could be used to specifically identify *A*. *healyi* and signify its potential for diagnosing GAE, though this needs to be validated using GAE tissue samples.

Although several new diagnostic methods for AK have been proposed, corneal scraping culture is still considered to be the gold standard [10,14,16]. One advantage of using the CE antibody-based diagnosis is its non-invasive nature. CE is a protein secreted by members of the *Acanthamoeba* [32] and as such, their presence in the conditioned media of various *Acanthamoeba* spp. have been confirmed in our study (Figs 1B and 5B). Based on this finding, AK diagnosis using the ocular secretions such as tears of AK patients could be feasible as CE proteins are expected to be present. Our ELISA results confirmed that the CE antibody is highly sensitive and can detect CE antigens at extremely low concentrations (Fig 2). However, it is possible that CE may only be present in trace amounts in ocular secretions, such as tears of AK patients. For this reason, the detection capability of the CE antibody needs further evaluation using clinical samples to validate the findings provided here.

In summary, we generated a polyclonal antibody against the secretory CE protein of *A*. *castellanii* that specifically detected *A*. *castellanii* trophozoites and cysts. The CE antibody also interacted with other 6 different members of *Acanthamoeba* spp. These results provide important information for developing an alternative diagnostic method for *Acanthamoeba* infection.

## Supporting information

S1 Raw images (PDF)
