# Supplementary material for: Detection of Acanthamoeba spp. using carboxylesterase antibody and its usage for diagnosing Acanthamoeba-keratitis
Source: PLoS One. 2022 Jan 5;17(1):e0262223. doi: 10.1371/journal.pone.0262223 (PMC8730387; doi:10.1371/journal.pone.0262223)
Supplement: S1 Raw images — (PDF) [file pone.0262223.s001.pdf]

Figure 1

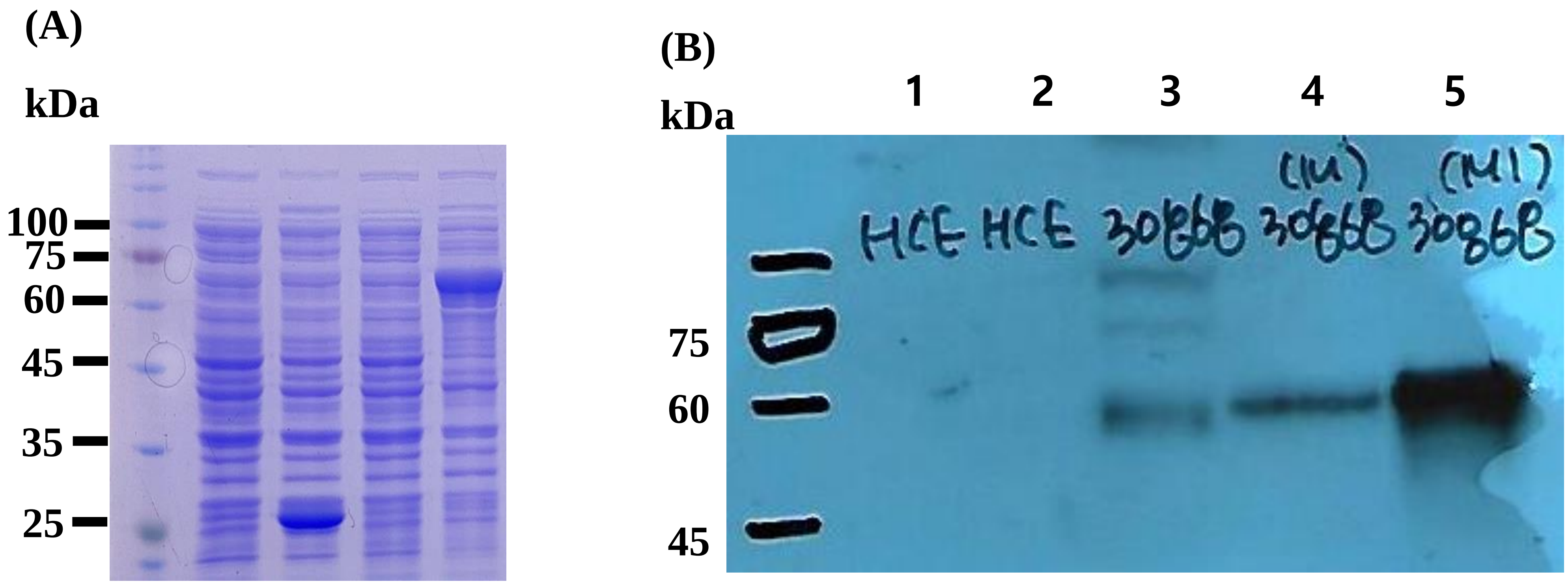

Lane 1: pGEX 4T-3 (- IPTG)
Lane 2: pGEX 4T-3 (+ IPTG)
Lane 3: pGEX 4T-3-CE (- IPTG)
Lane 4: pGEX 4T-3-CE (+ IPTG)

Lane 1: cell lysate of HCE cells
Lane 2: conditioned media of HCE cells
Lane 3: cell lysate of *A. castellanii* (50 ug)
Lane 4: conditioned media of *A. castellanii* (15 ug)
Lane 5: conditioned media of *A. castellanii* (50 ug)

Figure 5

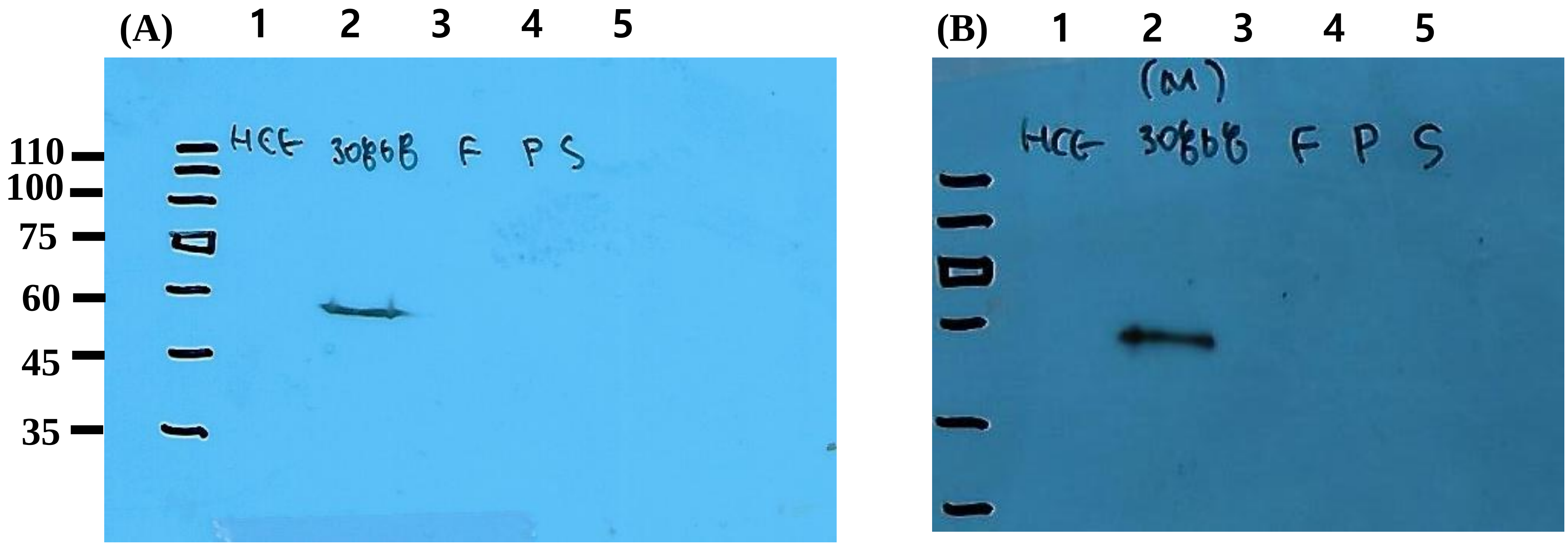

Lane 1: cell lysate of HCE cells
Lane 2: cell lysate of *A. castellanii*
Lane 3: cell lysate of *F. solani*
Lane 4: cell lysate of *S. aureus*
Lane 5: cell lysate of *P. aeruginosa*

Lane 1: conditioned media of HCE cells
Lane 2: conditioned media of *A. castellanii*
Lane 3: conditioned media of *F. solani*
Lane 4: conditioned media of *S. aureus*
Lane 5: conditioned media of *P. aeruginosa*
